# Impact of Metabolism on T-Cell Differentiation and Function and Cross Talk with Tumor Microenvironment

**DOI:** 10.3389/fimmu.2017.00270

**Published:** 2017-03-13

**Authors:** Soumaya Kouidhi, Amel Benammar Elgaaied, Salem Chouaib

**Affiliations:** ^1^ISBST, Laboratory BVBGR, LR11ES31, Higher Institute of Biotechnology of Sidi Thabet, University of Manouba, Sidi Thabet, Tunisia; ^2^Laboratory of Genetics, Immunology and Human Pathology, Faculty of Sciences of Tunis, University Tunis El Manar, Tunis, Tunisia; ^3^Institut National de la Santé et de la Recherche Médicale (INSERM) UMR1186, Laboratory «Integrative Tumor Immunology and Genetic Oncology», Equipe Labellisée LIGUE 2015, Villejuif, France; ^4^Institut National de la Santé et de la Recherche Médicale (INSERM), Gustave Roussy, University of Paris-Sud, Villejuif, France; ^5^Institut National de la Santé et de la Recherche Médicale (INSERM), Gustave Roussy, Université Paris-Saclay, Villejuif, France

**Keywords:** immune system, T-lymphocytes, tumor cell metabolism, cancer, hypoxia, tumor microenvironment

## Abstract

The immune system and metabolism are highly integrated and multilevel interactions between metabolic system and T lymphocyte signaling and fate exist. Accumulating evidence indicates that the regulation of nutrient uptake and utilization in T cells is critically important for the control of their differentiation and manipulating metabolic pathways in these cells can shape their function and survival. This review will discuss some potential cell metabolism pathways involved in shaping T lymphocyte function and differentiation. It will also describe show subsets of T cells have specific metabolic requirements and signaling pathways that contribute to their respective function. Examples showing the apparent similarity between cancer cell metabolism and T cells during activation are illustrated and finally some mechanisms being used by tumor microenvironment to orchestrate T-cell metabolic dysregulation and the subsequent emergence of immune suppression are discussed. We believe that targeting T-cell metabolism may provide an additional opportunity to manipulate T-cell function in the development of novel therapeutics.

## Introduction

It is well admitted that one of the mechanisms by which immune cells integrate the signals required for their proliferation, migration, differentiation, and effector functions is through the modulation of their metabolic activity (1). In this regard, T cells metabolically reprogram and upregulate glucose and amino acid, to allow the synthesis of the new macromolecules required for their proliferation and effector function (2, 3). Furthermore, beyond these key nutrients, iron uptake is also critical for T-cell function (4). Indeed, development and differentiation of antigen-specific T cells depend on iron uptake and internalization via type I transferrin receptor (5). Several previous studies suggested that iron deficiency impaired T-cell proliferation and cytokine production in activated T cells. Conversely, less is known about the effect of iron overload on T-cell function (6).

Nevertheless, how metabolism regulates immune T-cell differentiation, function, and plasticity remains very challenging and how immune cells function in terms of their intracellular metabolism and how these metabolic pathways affect the phenotype and activation of immune cells is attracting a lot of attention at present. Tumor progression is characterized by a tangled network of relationships among different cell types that collectively exploit a metabolic reprogramming and mutually influence their functionality and, in particular, T-cell functions. Our recent knowledge of T-cell molecules involved in the regulation of antitumor T-cell responses has led to the development of several monoclonal antibody-based therapies, against molecules like cytotoxic T-lymphocyte antigen (CTLA-4) or programmed death-1 (PD-1) (7). Although these treatments have shown unprecedented responses in some patients suffering from several cancers (8–10), the response rates are usually low and transient. This is likely due to multiple mechanisms suppressing antitumor immune functions within an unfavorable tumor milieu and metabolism. The metabolic activity of T cells in the context of tumor microenvironment could be one of the key mechanisms.

It should be noted that the dynamic and reciprocal interactions between tumor cells, metabolites, and a variety of cells including immune cells from the tumor microenvironment orchestrate several events, which are critical for tumor evolution toward metastasis. In this context, many cellular and molecular elements of the tumor ecosystem are emerging as attractive targets for therapeutic approaches. Among these targets, hypoxia, which is a hallmark of solid tumors, is strongly associated with advanced disease stage and poor clinical outcome. This is, in part, due to inappropriate local immune reaction and resistance of hypoxic tumor cells to cytotoxic treatments. In fact, most human tumors develop a pathophysiological microenvironment during growth, characterized by an irregular microvascular network and regions of chronically and transiently hypoxic cells. We and others provided evidence that hypoxia plays a crucial role in tumor promotion and immune escape by conferring tumor resistance (11) immunosuppression (12) and tumor heterogeneity (13), which contributes to the generation of diverse cancer invasion programs and enhanced stroma plasticity (11, 14). Therefore, it is of major interest to understand how immune cell intracellular metabolism and some metabolic pathways influence the acquisition of their phenotype, the regulation of their activation and effector function. The metabolic activity of T cells in the context of tumor microenvironment, its heterogeneity, and complexity is therefore an important consideration in immunotherapy. Clearly, if T cells play the music during an adaptive immune response, the metabolic tumor microenvironment calls the tune. Indeed, a better understanding of these metabolic related issues in relationship with T-cell activity may offer new therapeutic strategies in future to better control their plasticity and effector function and boost their efficacy and potential use in cancer immunotherapy approaches.

## Basic Overview of Metabolism in T Cells

Metabolism is the process whereby cells can either break down molecules to generate energy in the form of adenosine triphosphate (ATP) or synthesize several macromolecules. Metabolism could be divided into two complex pathways: the catabolic processes, critical for cellular proliferation and functions and the anabolic process, important for cellular growth.

Consistent studies focused on the molecular mechanisms that dictate metabolic reprogramming in the immune cells (15). It is now widely appreciated that T-cell metabolic remodeling plays a key role to shape immune response, in particular, antitumor immunity. Profound metabolic changes occur under tight regulation allowing T cells to maintain energy balance between anabolic and catabolic metabolism, which support adequate immune responses (16, 17).

**Figure 1:**
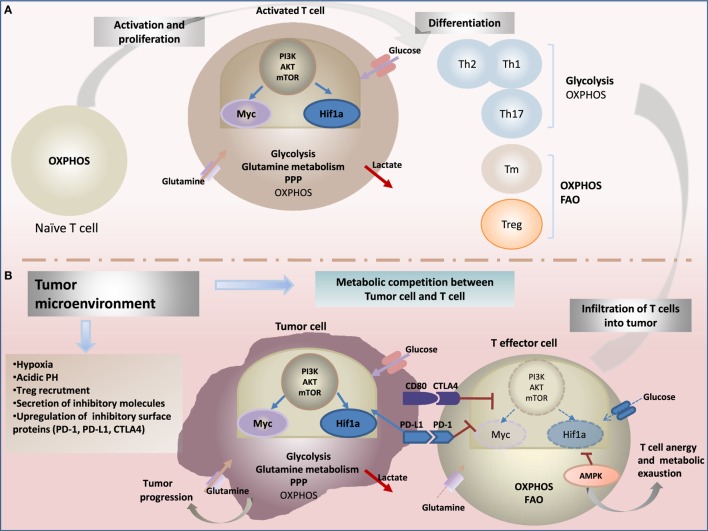
**Metabolic reprogramming drives: (A) T-cell fate and function and (B) antitumor-immune response**. **(A)** Upon activation through T-cell receptor (TCR) and costimulatory signals, T cells engage in growth, and differentiation into different cytotoxic, regulatory T cells (T_reg_), helper T (Th), and memory T (T_m_) subsets cells. Metabolic reprogramming has been shown to intimately support T-cell activation and differentiation. While naïve T cells rely on oxidative phosphorylation (OXPHOS) to maintain energy demand; activated T cells engage increased aerobic glycolysis and glutaminolysis consuming massive amount of glucose and glutamine, enabling to generate effector cytokines, including interferon-γ (IFN-γ) and IL-2. In contrast to cytotoxic and effector Th cells, the metabolic profile of T_reg_ and T_m_ cells rely on OXPHOS and fatty acid oxidation (FAO) to support their survival and differentiation. The central energy-monitoring system underlying this metabolic remodeling is the phosphoinositide 3-kinase/protein kinase B/mammalian target of rapamycin (PI3K/AKT/mTOR) pathway that boosts glycolytic activity in T cells via activation of transcription factors hypoxia-inducible factor-1α (HIF-1α) and Myc pathways. **(B)** Tumor-specific T cells are often rendered dysfunctional due to an immunosuppressive microenvironment. Infiltrating T cells are reprogrammed by the tumor favoring its survival and immune escape. Cancer cells induce several metabolic changes in the microenvironment. Tumor-mediated decreased extracellular nutrients levels cause impaired glycolysis capacity and IFN-γ production in T cells. Cancer cells also generate a hypoxic microenvironment. Hypoxia stabilizes HIF-1α and enhance glycolysis in tumor cells, a phenomenon recognized as “the Warburg effect.” HIF-1α also enhances constitutive expression of programmed death ligand-1 (PD-L1) leading to activation of Akt/mTOR pathway. Activating immune checkpoints and PD-L1 signaling through binding to its receptor programmed death-1 (PD-1) leads to dampening of the Akt-mTOR pathway and reduced T-cell glycolysis. Collectively, tumor environment affects metabolic fitness of infiltrating immune cells and drives impairment of antitumor effector function and increased tumor progression.

During quiescence, T cells require energy-oriented oxidative metabolism and relatively small amounts of glucose, amino acids and fatty acids to maintain basic energetic, primarily anabolic and minimal replacement biosynthesis demands. Encounter with cognate antigen activation, T-cell stimulation by T-cell receptor (TCR) ligation and binding with costimulatory molecules induce metabolic remodeling (18, 19). In fact, metabolism shifts to glycolysis to support rapid growth and to biosynthesis for differentiation into effector T cells (T_eff_) (1, 20, 21) (Figure 1A). Albeit, aerobic glycolysis is less efficient than oxidative phosphorylation (OXPHOS) at yielding ATP, it generates metabolic intermediates which are important for cell growth and proliferation as well as for cytotoxicity and cytokine production. Nevertheless, glycolytic pathway generates macromolecule precursors required in the pentose phosphate pathway (PPP) for cell growth and NAD phosphate (NADPH) production important for anabolic pathways and maintaining redox balance (22).

After pathogen clearance, most T cells go through apoptosis while few of them remain as long-lived memory cells responsible for enhanced immunity against upcoming pathogens or tumors re-exposure (23).

## Distinct Metabolic Programs for T Cells Differentiation and Function

T lymphocytes (T cells) that undergo an immune response constitute an ideal system to study the rapid shift from quiescent to active state that belongs to growth, proliferation, and differentiation into largely heterogeneous T-cell subsets. Emerging concepts in immunology suggest that lymphocyte activation is intricately linked to metabolic reprogramming (24–26). In fact, metabolism fundamentally underpins T-cell function and lymphocytes metabolism is dynamically regulated depending on their separate phases of development: (1) naïve or resting, (2) effector or activated, and (3) memory T (T_m_) cells (27).

Naïve T cells are activated to rapidly respond to foreign pathogens or inflammation through a tight interaction with the TCR and major-histocompatibility complex. Further, T cells enter the effector phase of rapid growth, proliferation, and differentiation. T_eff_ could be divided into cytolytic T cells, secreting granzyme B, perforin, interferon-γ (IFN-γ), into helper T cells (Th) including the type-1 (Th1), type-2 (Th2), and type-17 (Th17) producing characteristic cytokines or into regulatory T cells (T_reg_) (28, 29). T_eff_ play a pivotal role to mediate antitumor immunity. Hence, T_reg_ obstruct T_eff_ activity and suppress immunity, showing a poor prognosis in many cancers (30). After expansion phase and antigen clearance, most clonally differentiated T cells undergo apoptosis, while a small fraction become quiescent T_m_ cells, responsible for enhanced immunity after re-exposure to the pathogen (31, 32). The differences in functional and phenotypic characteristics of quiescent T cells and activated T cells are supported by differential metabolic requirements (17). Each subset of T-cell demonstrates unique metabolic demands and signaling pathways that contribute to its fate and function (25).

Quiescent T cells and activated T-cell fate are characterized by different metabolic pathways (33–35). Globally, activated T cells adopt an anabolic metabolism supporting rapid proliferation whereas quiescent T cells engage catabolic metabolism (36). T_eff_ subtypes switch their metabolic program to robust aerobic glycolysis, but increased glycolytic rates occurred much higher in Th1, Th2, and Th17 cells than in T_reg_ cells (24). T_reg_ cells sustain enhanced fatty acid oxidation (FAO) metabolism as a major source of energy to maintain their survival (37–39). Upon antigen encounter, upregulation of aerobic glycolysis in extensive proliferating T cells is accompanied with glutaminolysis, PPP, not only to support ATP generation, but also to enhance biosynthesis of crucial intermediates and precursors necessary for subsequent macromolecules that are incorporated into cellular biomass (40, 41) (Figure 1A). Th17 cells rely, in particular, on increased glycolysis. Hence, inhibiting glycolysis during Th17 cell differentiation re-enforce T_reg_ generation (42). Nevertheless, consistent data suggest that mitochondrial reactive oxygen species (ROS) produced during OXPHOS is also crucial to activate T-cell and to enhance antigen-specific proliferation. However, excessive ROS levels are toxic for T cells and leads to apoptosis (43). CD4 + regulatory T lineage cells exhibit a mixed metabolic program involving mainly FAO and OXPHOS and low level of glycolysis (44). T_reg_ favor FA catabolism via b-oxidation and prioritize oxidative ATP to meet their energetic demands, an important metabolic phenotype for the differentiation of T_reg_ (38).

After the clearance of pathogens, the remaining antigen-specific T cells (T_m_ cells) as a quiescent T-cell population share common metabolic requirements with other nonproliferating cells. T_m_ cells maintain catabolic profile with lower nutrient uptake and biomass synthesis and predominantly engage mitochondrial OXPHOS and FAO metabolism for long-term persistence, ATP production and the capacity to vigorously respond to antigen stimulation (45). Several studies revealed that maintaining mitochondrial mass is critical for T_m_ cells development since it offers the opportunity to use a wide range of substrates responsible for energy generation, like fatty acids (46, 47). FAO constitute a preferred fuel source for T_m_ cells as this lipid oxidation generates intermediate of tricarboxylic acid (TCA) cycle related to OXPHOS metabolism. However, their detailed metabolic profiles remain to be explored (25).

Metabolic regulation of T-cell fate and function involves a network of molecular regulators. The main induced signaling pathways underlying the activation through the TCR with CD28 costimulation, is the phosphoinositide 3-kinase/protein kinase B/mammalian target of rapamycin (PI3K/Akt/mTOR) (48–50). Increasing evidences suggest that the mammalian target of rapamycin (mTOR) is a central regulator of cell metabolism. Interestingly, T-cell differentiation to effectors or T_m_ cells is governed in part by asymmetric partitioning of fate determining proteins (51, 52). Recent study demonstrates that asymmetric division of T cells generates two sets of daughter cells with differential mTORC1 activity (53). The first set exhibits increased activity mTORC1, as well as high levels of glycolysis and effector molecules expression. Besides, the second T-cell set shows decrease in mTORC1 activity associated with enhanced rates of lipid metabolism and antiapoptotic molecules. Behind, the latter daughter cells display enhanced long-term survival and differentiate to T_m_ cells (53). This pathway plays key transcriptional and post-transcriptional roles to promote anabolic gene expression and intracellular trafficking of nutrient transporters (54). mTOR is the downstream target of the PI3K–AKT signaling and a central player governing metabolic reprogramming and fate of T-cell (55–57). Two major transcription factors are upregulated by mTOR: avian myelocytomatosis virus oncogene cellular homolog (c-Myc) and hypoxia-inducible factor-1α (HIF-1α) (58, 59) (Figure 1A). It has been shown that c-Myc is crucial to activate glucose transporters (GLUT) and key enzymes for enhancing glucose influx and glycolysis that accompany early stage of T-cell growth, proliferation, and the transition from a naïve T-cell to a T_eff_ cell (60). Furthermore, c-Myc is responsible for enhanced glutaminolysis by inducing glutamine transporters and glutaminase1 expression to sustain cell growth and proliferation (60, 61).

Hypoxia-inducible factor-1α is another master transcription factor monitoring glycolytic enzymes expression (62, 63). HIF-1α acts also to downregulate mitochondrial oxygen consumption and inhibit TCA cycle. At later times of differentiation, the role of HIF-1α appears more complex to mediate T-cell fate and function (64). HIF-1α is reported to play a more selective role in inflammatory Th17 CD4 T-cell subsets (42) and cytolytic CD8 T (58). In addition, HIF-1α appears to influence the balance of Th17:T_reg_ cells (65). Indeed, it directly promotes glycolysis in differentiating Th17 cells and reciprocally increases Th17 differentiation and decreases T_reg_ differentiation (66). However, *in vitro* and *in vivo* studies demonstrate that a lack of HIF-1α strongly impair Th17 cell development and drives T_reg_ cell differentiation and FAO. T_reg_ cells unlike other T_eff_ cells mainly display increased FAO metabolism and enhance AMP-activated protein kinase (AMPK) activation (67). The utilization of lipid oxidation by T_reg_ cells might play a central role in their survival advantage over T_eff_ cells and in the maintenance of a stable pool of pro-tumor (68, 69).

Finally, the mechanisms regulating the transition of T cells from effector to memory states remain to be elucidated. Recent studies demonstrated that mitochondrial FAO in T_m_ cells require stimulation of tumor necrosis factor receptor-associated factor 6 pathway (70). Further, memory CD8+ T-cell development is also supported by activating the energy sensor AMPK pathway (71, 72). FAO has clinical implications for memory CD8+ T as well as for T_reg_ cells (73). In fact, administration of metformin or the mTOR inhibitor rapamycin, reduce mTOR activity and induce AMPK phophorylation that in turn perform lipid oxidation and enhance the formation of T_m_ cells after infection and increase T_reg_ responses in asthma model (74, 75).

## Fueling T-Cell Proliferation

Increasing data suggest that regulation of metabolic fuels uptake is a critical component of T-cell activation to accomplish their functional requirements. Yet, limiting conditions could suppress the suitable access to nutrients, causing a barrier to T-cell function. To maintain a proper response, T-cell activation requires the upregulation of both glucose and amino acid transporters (1, 76). Several metabolic pathways that are imminent for lymphocyte proliferation are supported by the availability of these fuels (24).

### Glucose

Glucose is the most used nutrient predominantly existing in the surrounding environment, and glucose metabolism, in particular, is essential for T cells for normal survival and function. Glucose is a critical substrate for energy production, and its deprivation prevents T-cell function despite the presence of other alternative carbon source (77, 78). When T_eff_ are activated, glucose uptake raises to maintain aerobic glycolysis and subsequently to support growth and proliferation, whereas glucose use via OXPHOS is decreased (79). Further, the expression and trafficking patterns of GLUT are upregulated allowing T cells to enrich their intracellular glucose. The GLUT consists of 14 different members (GLUT1–14) relying on diverse substrate specificities (80). GLUT2 and GLUT3 are expressed in resting human peripheral blood T cells, while GLUT1 is expressed at a low level in naïve T cells, but rapidly induced upon T-cell activation. Consequently, overexpression of GLUT1 after TCR activation leads to increased glucose uptake and enhanced expression and activity of glycolytic enzymes. During glycolysis, glucose is not fully oxidized in the mitochondria but rather broken down into pyruvate that is converted into lactate even though in presence of sufficient oxygen (81). Glucose could be also derived toglucose-6phosphate and further directed into the PPP, providing precursors for the synthesis of nucleotides and aromatic amino acids (77).

It has also been reported that T-cell cytokine production is also relying on glucose. In fact, data showed enhanced T-cell cytokine production such as IL-2 and IFN-γ in transgenic model expressing GLUT1 specifically in T cells (78). In contrast, glucose deprivation has been shown to strongly inhibit cytokine production and to decrease cytolytic activity of CD8+ T cells, marked by reduced granzyme and perforin production. Thus, failure to properly upregulate glucose metabolism during T-cell activation can lead to impaired proliferation. As a consequence, T cells can enter to anergy if they survive this metabolic stress, or they die by apoptosis. Collectively, glucose is fundamental to support proliferation and effector functions that accompany clonal expansion of T_eff_. Besides, T_reg_ cells do not depend on high rates of glucose as they express low levels of GLUT1 and rely on lipid oxidation for energy (39).

### Glutamine

Glutamine is a nonessential amino acid and the most abundant nutrient in the blood. Glutamine constitutes also a critical substrate for T cells activation and growth process. Following T-cell activation through efficient TCR signaling, the uptake and biosynthesis of amino acids or amino acid transporter expression are dramatically increased (82, 83).

Glutamine catabolism is dramatically induced in active T cells providing intermediate molecules necessary for different pathways of biosynthesis and substrates for mitochondria (84, 85). During glutaminolysis, glutamine carbon backbone can be converted to α-ketoglutarate to maintain homeostasis of the TCA, or to lactate that generates NAD and NADPH (86). During T-cell activation glutamine can be used, providing pyruvates to overcome intense aerobic glycolysis levels (87). Further, activated T cells selectively increase glutamine uptake. This increase has been suggested to be concomitant with induced expression of glutamine transporters, recognized as members of the sodium-dependent neutral amino acid transporter (SNAT) family. In fact, the previous study demonstrates rapidly enhanced mRNA expression of SNAT1 and SNAT2 isoforms after *in vivo* stimulation of T cells (82). However, lack of glutamine can result in profound inhibition of cell growth, proliferation, and cytokine production (88). Since T-cell activation is strongly impacted by glutamine, thus different aspects of glutamine metabolism could serve as novel targets for immune modulation.

### Tryptophan and Arginine

In addition to glutamine, other limiting amino acids such as tryptophan and arginine have been suggested to be crucial for T-cell activation and function. This concept has gained interest especially in cancer context, where tumor-induced extracellular depletion of these amino acids alters T-cell activity and causes their anergy. Tryptophan is an essential amino acid required for the production of several important molecules and its catabolism through the kynurenine pathway generate metabolites such as kynurenine, kynurenic acid, 3-hydroxy-kynurenine, and 3-hydroxy-anthranilic acid (89). Numerous studies showed that tryptophan plays a key role in T-cell survival and activation whereas its metabolites eliminate T-cell function and are able to induce T-cell apoptosis (90). T_eff_ are affected by the decrease in tryptophan concentrations and high rates of toxic tryptophan-metabolites induced by mature antigen-presenting cells expressing enzymes that catabolize tryptophan (91). Tryptophan degradation is one of a resistance mechanisms adopted by tumors to avoid immune suppression (92, 93). Three enzymes were identified to control tryptophan degradation through the kynurenine pathway: tryptophan-2,3-dioxygenase, indoleamine 2,3-dioxygenase 1, and indoleamine 2,3-dioxygenase. Hence, T-cell cycle progression is prevented and T_eff_ cells shift to anergy and apoptosis. In hostile tumor microenvironment context, such inhibition is resulting in suppression of antitumor immune responses (94).

In addition to tryptophan, arginine has gained much attention as an important amino acid in T-cell function. Arginine is a versatile amino acid engaged in protein synthesis and in generating many metabolites precursors including, polyamines, and nitric oxide involved in immunometabolism (95). Indeed, deficiency in extracellular arginine or in enzymes responsible of *de novo* synthesizing arginine [argininosuccinate 1 (ASS1)], has been found to critical during activation (96). Low levels of arginine impair T-cell proliferation, aerobic glycolysis and reduce cytokine production and expression of activation markers such as CD25 and CD28 (97, 98). Further, deletion of ASS1 blunt *in vitro* Th1 and Th17 cell polarization, even in the presence of extracellular arginine (99). Interestingly, recent study showed that increased arginine levels display improved survival capacity of T memory cells and antitumor activity (95). Taken together and according to the beneficial effect of arginine and tryptophan on T-cell metabolic adaptation and antitumor activity, both amino acids would be exploited as an attractive target for therapeutic intervention in antitumor response (96).

## Warburg Effect or How Cancer Cell Rewire Metabolic Program

It is well established that cancer cells must reprogram cellular pathways to enable their growth and proliferation. Tumor cells reprogram their metabolic pathways and rely upon increased glucose uptake and high rate lactate production, principally through aerobic glycolysis (100), regardless of the level of oxygen (101). Metabolic switch of cancer cell supports biosynthesis of essential macromolecules (nucleic acids, lipids, and amino acids), through interconnected pathways. This metabolic program was recognized since 1920s by Otto Warburg as the “Warburg effect” (102, 103), a strategic metabolic adaptation enhancing rapid tumor growth, proliferation, and to dampen antitumor immunity, thus representing one additional hallmark of cancers. Since 1923, Otto Warburg has reported that cancer cells acquire irreversible switch of their energy-producing machinery from mitochondrial OXPHOS respiration, to aerobic glycolysis (104). Glycolysis is a predominant energy source for cancer cells, occurring either under aerobic or hypoxic conditions to produce large amounts of lactate, and much less efficient than OXPHOS for producing ATP (105, 106) (Figure 1B). This reprogramming of cancer cell metabolism has been acknowledged recently as a hallmark of cancer with many faces (107, 108). By analogy to immune cells, similar metabolic features with T cells during activation are observed. But, despite an apparent similarity, there is deep down a wide difference between glycolysis in activated T cells and cancer cells. Such metabolic transitions in T cells are part of a physiological adaptation process. However, intrinsic genetic mutations and external responses to the tumor microenvironment monitor the metabolic phenotype of tumor cells (109, 110). Cellular dysregulation of oncogenic signaling pathways are the result of the loss of tumor suppressors (such as p53) or the activation of oncoproteins (such as PI3K) (111). As a consequence, cancer cells thereby gain selective growth and survival (112).

Cancer cells use the Warburg effect as strategic metabolic adaptation to satisfy their urgent requirements for growth and proliferation under tumor microenvironmental limitations for oxygen and nutrients (113, 114). Under hypoxic conditions, cancer cells accelerate metabolism that lead to increased NADPH rate to cope with higher ROS levels (115, 116). Thus, the Warburg effect also supports tightly controlled redox balance for cancer cells, considered as important survival mechanism (117).

Glucose is considered as prominent player in the alterations of metabolism and energetic of cancer cells (118). Increased glucose uptake lead to upregulated glycolysis and thus more pyruvate is produced even in normoxia conditions. Under limited oxygen availability (hypoxia), more pyruvate avoids TCA cycle and generates excess of lactate secreted thereby in the tumor microenvironment (118, 119). In addition to its central role as a carbohydrate nutrient for ATP synthesis, new evidence revealed that high glucose uptake is also important for biomass synthesis needed for rapidly proliferating cancer cells. Upregulation of glycolysis increased several metabolic intermediates that may be shunted to interconnected pathways, as PPP (120, 121). The resulting glycolytic intermediates such fructose-6-phosphate, glyceraldehyde-3-phosphate, and 3-phosphoglycerate are critical for *de novo* synthesis of ribonucleotides, amino acids, and phospholipids, respectively (122).

Glutamine is the most abundant free amino acid and essential source of carbohydrate for proliferating cells. Cancer cells display increased glutamine demand and consumption. Interestingly, the glutamine dependence extends beyond protein synthesis to other important requirements (123). Rapidly proliferating, cancer cells use glutamine to fuel biosynthesis of nucleotides, to replenish TCA cycle intermediates through a process called anaplerosis, or to be taken from the mitochondria and then modified into lactate (glutaminolysis) (124, 125). Glutamine metabolism occurs in cancer cells, in general, with concomitant production of NADPH that not only maintains cellular redox but also reduces agent in varied biosynthetic pathways–underlying *de novo* fatty acid synthesis (126).

The molecular drivers that lead to the shift of cancer cell from oxidative to glycolytic metabolism are distinct and tend to happen simultaneously. Cancer metabolism adaptation to the anabolic program has been suggested to be under direct management by various transcription factors, such as Myc and hypoxia-inducible factor 1 (HIF-1) (127, 128).

Myc is a transcription factor upregulated in tumors and considered as master regulator of normoxic cancer cell reprogramming (129). Indeed, Myc contributes to cancer cells switch to aerobic metabolism by facilitating cellular glucose uptake and activating the expression of numerous genes essential for glycolysis. Furthermore, Myc plays important role to promote macromolecules synthesis and mitochondrial biogenesis, critical for fast developing cancer cells (130, 131).

Upon rapid proliferation, hypoxia becomes a key mediator of the Warburg effect and a common feature of human tumors. Extensive studies have provided evidence that cancer cells utilize hypoxia as physiological adaptation pathway that promotes metabolic changes in fast growing tumors (132). Indeed, under hypoxic tumor microenvironment, the uptake of glucose and the glycolytic flux are increased. This metabolic adaptation is mainly orchestrated through the upregulation of the transcription factor, HIF-1α. HIF-1α is induced by low oxygen conditions and recognized as independent marker of poor prognosis (133, 134). The activated tumor glycolytic flux involving HIF-1α implies upregulation and increased activity of several glycolytic protein including key glycolytic enzymes (HK2, PFK-L, PKM2, and LDH-A) and GLUT (GLUT1 and GLUT3) (135, 136). In contrast to Myc, HIF-1 strongly inhibits mitochondrial respiration and biogenesis (111).

Furthermore, PI3K/Akt/mTOR is one of the most frequently altered signaling pathway known to play an important role in glycolysis, cancer metabolism and cancer cell proliferation (137, 138) (Figure 1B). It is well known that this pathway is activated under the loss of function of the tumor suppressor gene phosphatqase and tensin homolog. The best studied driver of tumor glycolytic program in such pathway. The latter has been reported to induce GLUT expression and to stimulate phosphorylation of key glycolytic enzymes (139). In addition, AKT1 strongly activates mTOR signaling pathway. Hence, mTOR is constitutively activated during tumorigenesis (140) and constitutes a key metabolic issue, coupling cell growth to protein, and lipid biosynthesis (141).

## Tumor Microenvironment Abrogates T-Cell Metabolic and Immune Checkpoints

Immuno-metabolism plays a key role of adaptive immunity and is particularly central to effective antitumor T-cell responses. T cells, following the metabolic strategies of growing tumors, have to start their effector programs. However, most of human tumors proliferate in spite of the presence of tumor associated antigen-specific T cells. In fact, tumor microenvironment may impose several limitations to dampen T-cell immunity (142) and deplete crucial nutrient availability and handling, such as glucose or amino acids (143). It can also stimulate conserved negative feedback mechanisms, such as through PD-1 (144). Besides, tumor cells must evade the checkpoint controls under such stressful metabolic conditions.

Tumor microenvironment is a forbidding environment that can pose significant metabolic challenges for infiltrating T cells to impair the effectiveness T-cell response. It is likely that T cells undergo immune suppressive networks that impair their specific functions and thereby enable tumor escape (145, 146). Many different molecular and cellular mechanisms have been proposed to contribute to the failure of T cells in tumor eradication. Recent studies have started to reveal that the feature and function of T_eff_ in tumors are severely influenced by the tumor microenvironment context (147). Indeed, tumor microenvironment components form a very complex immunosuppressive network in cancer (148), lead to metabolic and immune checkpoints abrogation, which limits T-cell activation and induces T-cell dysfunction (149, 150). However, the exact mechanisms remain insufficiently understood.

Evidence is beginning to emerge suggesting that alterations of the T-cell metabolic pathways are critical to impair antitumor immunity, supporting immune escape (151). Cancer cells are recognized to be the most important players in tumor microenvironment mediating immune suppression. In fact, metabolic interplay and nutrient (glucose and glutamine) competition between cancer cells and T cells exist. Such competition is recognized as a key driver of cancer progression (152, 153). Due to high demand for energy and increased glucose addiction and glycolysis rate, fast growing cancer cells consumes most nutrients and specifically increases rate of glucose intake, from the surrounding environment (154). As a consequence, tumor-imposed metabolic restrictions can mediate T-cell hypo-responsiveness during cancer. T cells dramatically reduced glycolysis and become unable to produce cytokines and to develop into tumor-specific T_eff_ cells, leading to a state of anergy (155) (Figure 1B). Thus, T_reg_ cells differentiation is favored to inhibit antitumor immune response, instead of expansion of tumor-specific T cells (156, 157). As a contrast to T_eff_ that suffer from a hostile tumor microenvironment, T_reg_ cells, feel comfortable with a similar environment (158). This is possibly the result of to the flow in growth factors (such as transforming growth factor-β) and chemokines (such as CCL22) promoting T_reg_ differentiation and recruitment (156, 159). One molecular explanation is that alteration of functional fate of T cells due to nutrient limitation could occur through modulation of metabolically sensitive signaling pathways. Under tumoral context, the balance between T_eff_ and T_reg_ may be directly disturbed when AMPK signaling pathway inhibits mTORC (56, 160). Opposing to mTORC, AMPK is activated in conditions where nutrients are limiting and promote oxidative metabolism (161) (Figure 1B). AMPK can be highly phosphorylated and activated in T_reg_. Consequently, T_eff_ function is impaired while T_reg_ cells are promoted. Furthermore, T_reg_ cells have also been reported to be induced under hypoxic tumor microenvironment, through over activated HIF-1α (12, 162). The presence of T_reg_ in solid tumors essentially correlates with poor prognosis (27).

Immunosuppressive tumor microenvironment is also characterized by elevated rates of ROS (115). Besides cancer cells, tumor-infiltrating leukocytes, including myeloid-derived suppressor cells, tumor-associated macrophages, and T_reg_, also generate excessive ROS (163). It has been demonstrated that high level of ROS in the tumor microenvironment downregulates T-cell activity and enhanced T-cell apoptosis, inhibiting subsequently antitumor immune response (164). However, although high levels of ROS impair T-cell metabolism and function, ROS at a low or moderate-concentration is indispensable for T-cell activation and effector function (165). Considering the paradoxal effect of ROS on T-cell function a tight balance between production and consumption of ROS should be accomplished to potentiate antitumor activity compromising T_eff_ function.

Under immunosuppressive tumor microenvironment T cells acquire an “exhausted” phenotype highlighted by upregulation of inhibitory receptors. Interestingly, to eradicate effectiveness of antitumor immune response, tumor hostile environment act not only to impair metabolic checkpoints of T_eff_ cells encountering tumor antigens, but also to abrogate immune checkpoints. Indeed, several negative feedback mechanisms are stimulated, such as PD-1 and CTLA4 pathways (166, 167), which can both promote T cells exhaustion (Figure 1B). Hence, further research is needed to identify new target to reverse exhaustion in addition to PD-1 and CTLA4.

Programmed death-1 is the major inhibitory receptor in T cells regulating T-cell exhaustion. Interaction of PD-1 with its ligand programmed death ligand-1 (PD-L1), allows the tumor to evade immune system by inhibiting T-cell function (168, 169). Recently, it has been reported that upon ligation, T cells receiving PD-1 signals can lower the capacity of T cells to express GLUT1, uptake glucose, and become unable to engage in glycolysis, glutaminolysis, or metabolism of branched-chain amino acids (144). Interestingly, PD-1 displayed an increased rate of FAO of endogenous lipids, and lipolysis is indicated by elevation of the lipase ATGL and by release of fatty acids (144). PD-1 signaling is associated with reduced cMyc expression and inhibition of activity of the PI3K/Akt/mTOR pathway, necessary for effector function (50, 170, 171). Besides, PD-L1 directly regulates tumor metabolism. Surface expressed PD-L1 is important for Akt/mTOR signaling to promote mTOR activity and glycolytic metabolism in tumor cells (172, 173).

Nonetheless, CTLA4 signaling also plays a key role in tumor immune escape since it inhibits CD28-mediated costimulation of T_eff_ and favors T_reg_ expansion (174, 175). Subsequently, CTLA4 may broadly impair T_eff_ cell activation against antigenic stimulation in part by reducing the capability of Akt to enhance GLUT1 expression, glucose uptake, and aerobic glycolysis, but without enhanced FAO as for PD-1 pathway (151).

## Immune Checkpoints Targeting for Enhancing T-Cell Function: Relationship with Metabolism

Metabolic reprogramming plays a pivotal role for appropriate T-cell activation that supports antitumor immunity. However, T-cell function is compromised by the immunosuppressive tumor microenvironment. Nevertheless, multiple mechanisms that instruct the development of immune suppression may exist to prevent effective antitumor response, but remain largely unclear. Metabolic and functional pathways in T cells may uncover new targets and challenges for cancer therapy (176). Therefore, manipulating metabolism may be a way to beneficially enhance or temper antitumor immunity. Current attractive therapeutic approaches which specially target T-cell metabolism are meant to use immunotherapy directed against several negative immunologic regulators CTLA-4 and PD-1/PD-L1 pathway (177–179).

In recent studies, it has been reported that mice exhibiting or transplanted with tumors were treated with checkpoint blockade therapy, such blockade increased the glucose concentrations in the extracellular tumor milieu and TILs from these mice had increased glucose uptake, glycolytic rates, activated mTORC1 pathway, and IFN-γ production (152). The same effects were reported after PD-L1 blockade or RNA interference directed against PD-L1 in cultured tumor cells (152). More importantly, T cells in allogeneic PD-L1^−/−^ bone marrow transplant recipients had elevated levels of GLUT1 and lactate production, suggesting a normal *in vivo* role for PD-1 signaling to restrain T-cell glucose metabolism (180).

Although there is a promising efficacy of immunotherapy, the clinical benefit has been restricted by tumor-derived immunosuppression and its related coinhibitory signals. Indeed, to escape antitumor immune response, tumors develop different strategies including secretion of immunosuppressive cytokines and chemokines (TGF-β, IL-10, VEGF, CCL2, and CCL12) (181) or immunosuppressive converting tryptophan and arginine enzymes [indoleamine-2,3-dioxygenase (IDO) and arginase, respectively] (98, 182, 183). In light of this, it would be reasonable to combine immunotherapy with an immunosuppression-blocking protocol. In particular, IDO is an attractive area for exploitation to potentiate immunotherapy, since it is highly expressed in the microenvironments of various tumors (184). Recently, preclinical studies demonstrate the efficiency of two IDO inhibitors to attenuate tumor growth (185, 186). Currently, IDO inhibitors entered clinical trials (187). Interestingly, *in vivo* study has been conducted on mouse melanoma model where synergistic immunotherapy strategy that locally targets PD-1 and IDO for the treatment of melanoma has been developed. The preliminary results are quite encouraging and showed enhanced T_eff_ cells and antitumor efficacy (188).

Therefore, the use of metabolism-targeting drugs working with checkpoint inhibitors might not only change the activation and differentiation program of tumor-specific T cells but also prohibit the generation of exhausted T cells. Currently, there is a lack of data taking into consideration the metabolic consequences occurring in T cells and/or tumor cells by targeting these immune checkpoint pathways. Nevertheless, combined immunotherapeutic strategies would be exciting and show promise to improve the anticancer efficacy of immunotherapy in the future.

## Concluding Remarks

It is widely admitted that tumors are not autonomous masses of cells but function as organs composed of many interdependent cells supporting malignant cell survival, growth and progression. To ensure tumor growth and immune evasion, the tumor stromal components undergo numerous metabolic adaptations, reprogramming the mode of energy generation. T cells play key role in the orchestration of the immune response and T-cell metabolic adaptation acts as crucial checkpoint hijacked by tumors to dampen antitumor immunity as T cells are rendered dysfunctional, unable to carry out their effector functions. Accumulating evidence indicate that the diverse functions of the immune system require several bioenergetic processes and that T-cell metabolic reprograming relies upon the activation of distinct transcriptional and signaling pathways. In the context of tumor microenvironment, tumors impose several limitations to dampen T-cell immunity as T cells, experiencing the metabolic framework of growing tumors, fail to activate distinct pathways to accomplish their functional requirements. Tumor microenvironmental hypoxia is in this regard a relevant example demonstrating how the tumor microenvironment of a tumor can paralyze and neutralize T-cell functions. In fact, O_2_ is a master regulator of the CD8+ T-cell response and T lymphocytes face pathologically low O_2_ tensions within the tumor bed at which they will have to function. It has become clear that tumor-imposed metabolic restrictions may result in an impairment of T-cell function and that either some programmed changes or pathologic manifestations can inhibit the required energy essential for their several functions. Accordingly, attempts are made to identify approaches aiming at manipulating the reprogramming of T-cell metabolic pathways for therapeutic purposes, in particular, antitumor immunity.

## Author Contributions

All authors listed have made substantial, direct, and intellectual contribution to the work and approved it for publication.

## Conflict of Interest Statement

The authors declare that the research was conducted in the absence of any commercial or financial relationships that could be construed as a potential conflict of interest.

## Abbreviations

AKT, serine/threonine-specific protein kinase; AMPK, AMP-activated protein kinase; ATP, adenosine triphosphate; CTLs, cytolytic T cells; CTLA-4, cytotoxic T-lymphocyte antigen; FAO, fatty acid oxidation; GLUT, glucose transporters; HIF, hypoxia-inducible factor; IFN-γ, interferon-γ; MHC, major-histocompatibility complex; mTOR, mammalian target of rapamycin; c-Myc, avian myelocytomatosis virus oncogene cellular homolog; OXPHOS, oxidative phosphorylation; PD-1, programmed death-1; PD-L1, programmed death ligand-1; PI3K, phosphatidylinositol-3 kinase; PPP, pentose phosphate pathway; PTEN, phosphatqase and tensin homolog; ROS, reactive oxygen species; TAA, tumor associated antigen; TCA, tricarboxylic acid; TCR, T-cell receptor; T_eff_, effector T cells; Th, helper T cells; TNF-α, tumor necrosis factor; T_reg_, regulatory T cells.
